# First draft genome assembly of the Argane tree ( *Argania spinosa*)

**DOI:** 10.12688/f1000research.15719.2

**Published:** 2020-05-04

**Authors:** Slimane Khayi, Nour Elhouda Azza, Fatima Gaboun, Stacy Pirro, Oussama Badad, M. Gonzalo Claros, David A. Lightfoot, Turgay Unver, Bouchra Chaouni, Redouane Merrouch, Bouchra Rahim, Soumaya Essayeh, Matike Ganoudi, Rabha Abdelwahd, Ghizlane Diria, Meriem Alaoui Mdarhi, Mustapha Labhilili, Driss Iraqi, Jamila Mouhaddab, Hayat Sedrati, Majid Memari, Noureddine Hamamouch, Juan de Dios Alché, Noureddine Boukhatem, Rachid Mrabet, Rachid Dahan, Adelkhaleq Legssyer, Mohamed Khalfaoui, Mohamed Badraoui, Yves Van de Peer, Tatiana Tatusova, Abdelhamid El Mousadik, Rachid Mentag, Hassan Ghazal

**Affiliations:** 1 Research Computing and Cyber infrastructure, Computer Science Department, Southern Illinois University, Carbondale, IL, 62901, USA; 2 Polydisciplinary Faculty, Sultan Moulay Slimane University, Beni-Mellal, Morocco; 3 Estación Experimental del Zaidín, Consejo Superior de Investigaciones Científicas (CSIC), Granada, Spain; 4 Department of Plant Biotechnology and Bioinformatics, Ghent University, Ghent, B-9052 Ghent, Belgium, Belgium; 5 VIB Center for Plant Systems Biology, Technologiepark 927, Ghent, B-9052, Belgium; 6 Department of Genetics, Genomics Research Institute, University of Pretoria, Pretoria, 0028, South Africa; 7 National Center for Biotechnology Information, National Institutes of Health, Bethesda, MD, 20817, USA; 8 Biotechnology Unit, National Institute of Agricultural Research (INRA), Rabat, Morocco, Morocco; 9 Laboratory of Biotechnology and Valorization of Natural Resources (LBVRN), Faculty of Sciences, University Ibn Zohr, Agadir, Morocco, Agadir, Morocco; 10 Laboratory of Physiology, Genetics & Ethnopharmacology (LPGE), Faculty of Sciences, University Mohamed Premier, Oujda, Morocco; 11 IRIDIAN GENOMES. INC., Bethesda, MD, 20817, USA; 12 Department of Plant, Soil and Agricultural Systems, Southern Illinois University, Carbondale, IL, 62901, USA; 13 Laboratory of Plant Physiology, Faculty of Sciences, University Mohamed V in Rabat, Rabat, 10000, Morocco; 14 Department of Molecular Biology and Biochemistry, and Plataforma Andaluza de Bioinformática, University of Malaga, Malaga, Spain; 15 International Biomedicine and Genome Institute (iBG-izmir), Dokuz Eylül University, Current address: Egitim Mah. Ekrem Guer Sok. 26/3 Balcova, Izmir, Turkey; 16 National Center for Scientific and Technological Research (CNRST), Rabat, Morocco; 17 Polydisciplinary Faculty of Nador, University Mohamed Premier, Nador, Morocco; 18 National School of Computer Sciences & Systems Analysis, University Mohammed V in Rabat, Rabat, Morocco

**Keywords:** Argane, Argania spinosa, Endemic, Genome, Assembly, Morocco, International Argane Genome Consortium

## Abstract

**Background: **The Argane tree ( *Argania spinosa *L. Skeels) is an endemic tree of mid-western Morocco that plays an important socioeconomic and ecologic role for a dense human population in an arid zone. Several studies confirmed the importance of this species as a food and feed source and as a resource for both pharmaceutical and cosmetic compounds. Unfortunately, the argane tree ecosystem is facing significant threats from environmental changes (global warming, over-population) and over-exploitation. Limited research has been conducted, however, on argane tree genetics and genomics, which hinders its conservation and genetic improvement.

**Methods: **Here, we present a draft genome assembly of *A. spinosa*. A reliable reference genome of *A. spinosa* was created using a hybrid *de novo* assembly approach combining short and long sequencing reads.

**Results: **In total, 144 Gb Illumina HiSeq reads and 7.6 Gb PacBio reads were produced and assembled. The final draft genome comprises 75 327 scaffolds totaling 671 Mb with an N50 of 49 916 kb. The draft assembly is close to the genome size estimated by *k*-mers distribution and covers 89% of complete and 4.3 % of partial *Arabidopsis* orthologous groups in BUSCO.

**Conclusion: **The *A. spinosa* genome will be useful for assessing biodiversity leading to efficient conservation of this endangered endemic tree. Furthermore, the genome may enable genome-assisted cultivar breeding, and provide a better understanding of important metabolic pathways and their underlying genes for both cosmetic and pharmacological.

## Introduction

*Argania spinosa* (L. Skeels) is a tree endemic to the Middle West of Morocco and occupying arid and semi-arid regions totaling up to around 900,000 ha ^1^. The argane tree forest was recognized as biosphere reserve (Arganeraie Biosphere Reserve) by UNESCO in 1998 ^2^. It is the only unique member of the tropical Sapotaceae family in Morocco ^3^. In addition to its ecological role in preventing soil erosion and desertification, the argane tree has great cultural and socio-economic importance. The oil extracted from the seed is considered the most expensive edible oil in the world with great cosmetic value and therapeutic potential ^4– 6^. Argane oil represents a significant source of dietary fatty acids, while the Argane fruit is used as livestock feed by the local population ^7– 9^. Phytochemical composition of Argane fruits reveals different classes of bioactive compounds, including essential oils, fatty acids, triacylglycerols, flavonoids and their acylglycosyl derivatives, monophenols, phenolic acids, cinnamic acids, saponins, triterpenes, phytosterols, ubiquinone, melatonin, new aminophenols, and vitamin E. Argane oil contains high levels of antioxidant compounds. The long-chain fatty acids in Argane oil are primarily represented by unsaturated oleic acid, then linoleic acid, palmitic acid and stearic acid ^10^.

The distribution area of the Argane forest decreased drastically during the 18th century. Furthermore, about 44 % of the forest was again lost between 1970 and 2007. While there are multiple causes, desertification and overgrazing form the main pressures on the Argane forest ^11, 12^. Therefore, the management and conservation of the remaining genetic resources of Argane forest are urgent priorities. In recent decades, several studies have been conducted to evaluate the genetic diversity of the Argane tree using morphological ^13, 14^, chemical ^10, 15^, biochemical ^6, 16^ and standard molecular marker techniques, all with the aim of describing the genetic diversity of Argane trees and addressing ecological and conservation issues ^17– 25^. The karyotype of *A. spinosa* (L.) is constituted of ten pairs of chromosomes (2n =2x =20) ^3^. Until now, no reference genome was available of the *A. spinosa* species. Here, we present the Argane tree genome assembled from short and long DNA reads using a hybrid assembly strategy.

## Methods and results

### Plant material

**Figure 1.**
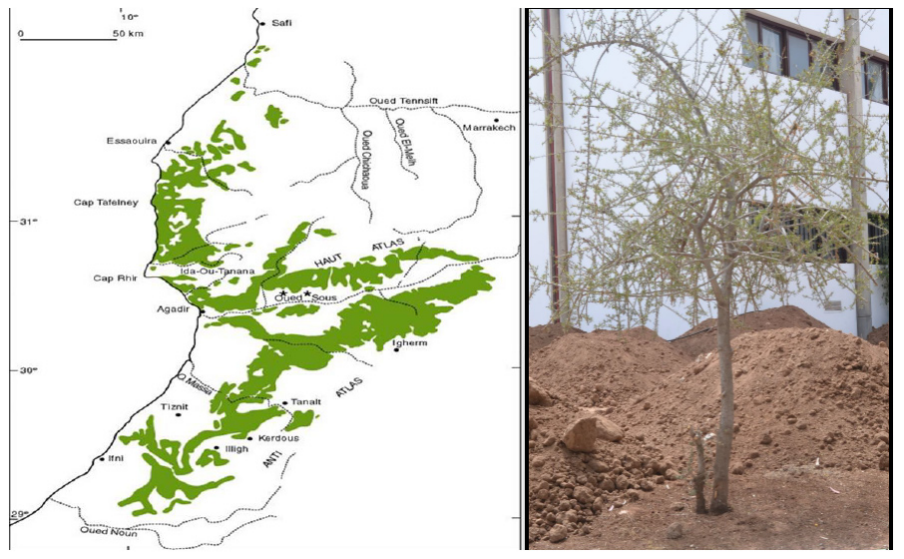
Information on the *Argania.spinosa* individual named # *Argane Amghar* whose DNA has been sequenced. ( **A**) Map of Sous region with *Argania* species distribution. ( **B**) Picture of the tree # *Argane Amghar*. (Photograph taken by A. El Mousadik).

The Argane tree ( *Argania spinosa*, taxid 85883, Sapotaceae, family, order Ericales), named *Argane AMGHAR*, to be sequenced was selected for its biological and ecological characteristics ( Figure 1). This was a 9-year-old shrub, with weeping form (geotropic, unlike the erect have) with only one main trunk 3 m in height. The ripe fruits has a rounded shape. The plant had semi-evergreen dwarf leaves. The shrub is native to the valley of the plain of Sous, an arid climate with an annual average rainfall of around 220 mm, located between the hills of the Anti-Atlas towards the South East, the Western High Atlas towards the North-West and the Atlantic Ocean towards the West (9°32′ 00″N, 30°24′ 00″W; Altitude: 126 m).

### DNA sequencing and data description

Genomic DNA was extracted from lyophilized leaf tissues of a single tree ( *Argane AMGHAR*) using the Plant DNeasy mini kit (Qiagen, USA). The Argane tree genome was shotgun-sequenced using both Pacbio ^TM^ (Menlo Park, CA, USA) and Illumina ^TM^ (San Diego, CA, USA) sequencing technologies, generating 7.6 Gb and 144 Gb of data, respectively. Paired-end libraries with average insert sizes of 600 bp were constructed with Nextera ^TM^ DNA Library Prep Kit for Illumina (New England BiolabsTM, New Brunswick, MA, USA). These libraries were sequenced on an Illumina HiSeq XTen platform using the PE-150 module and yielded 957,451,810 reads ( Table 1). These data were trimmed of adapters, yielding a clean set of 936,053,040 reads, representing 236× genome coverage, assuming a genome size of 573 Mb as estimated by the *k-mer* frequency analysis (described below). Raw reads were deposited at the NCBI Sequence Read Archive (SRA) under accession numbers: SRX3207155 and SRX3207156, corresponding to two independent runs from the same plant DNA sample. In addition, single-molecule long reads from the PacBio RS II platform (Pacific Biosciences, USA) were used to assist the subsequent *de novo* genome assembly using Illumina. Genomic sequencing libraries were constructed using the PacBio DNA template preparation kit 2.0 (Pacific Biosciences of California, Inc., Menlo Park, CA) for SMRT sequencing on the PacBio RS II machine (Pacific Biosciences of California, Inc.) according to the manufacturer’s instructions, with a size range of 2-15 kb. The constructed libraries were sequenced on six SMRT cells on a PacBio RSII sequencer. The sequences of the 6 SMRT cell runs were deposited at the NCBI SRA under accession numbers: SRX1898029/ SRX1898030/ SRX1898031/ SRX1898032/ SRX1898033/ SRX1898034. The sequencing runs produced about 7.6 Gb, consisting of 6,705,437 reads with an average read length of 2.5 kb and representing about 13× genome coverage, again assuming a genome size of 573 Mb ( Table 1).

**Table 1.** Summary of reads generated from genome sequencing and used in the assembly.

Library technology	Raw data		Trimmed data	
	Number of reads	Number of bases	Number of reads	Number of bases
Illumina HiSeq X Ten	957,451,810	144,575,223,310	936,053,040	135,539,587,270
PacBio RS II	6,705,437	7,649,825,228	1,910,887	7,078,584,472

### *In silico* genome size estimation

**Figure 2.**
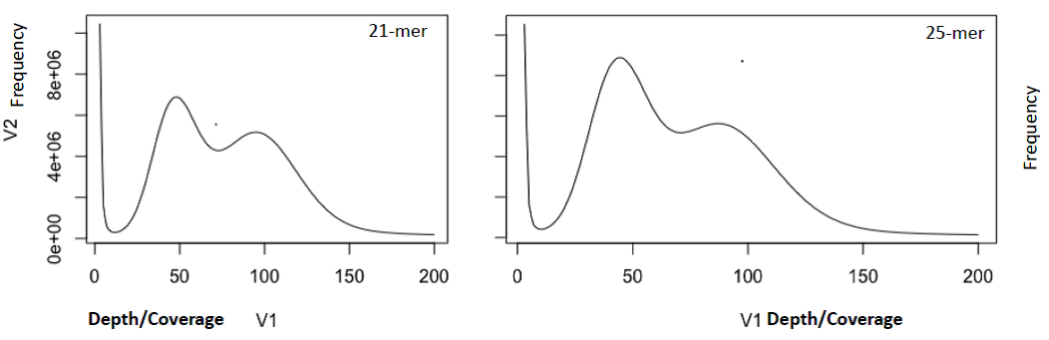
Distribution of 21 and 25-mers using Jellyfish with PE data Argane whole genome sequencing.

Trimmed reads from the Illumina platform were subjected to *k*-mer frequency distribution analysis with JELLYFISH v2.1.4 software ^26, 27^. Analysis parameters were set at - *k* 21 and 25, and the final result was plotted as a frequency graph ( Figure 2). Two distinctive modes were observed from the distribution curve: the higher peak at a depth of 44 and reflecting the high heterozygosity of the Argane genome; the lower peak provided a peak depth of 87 for the estimation of the genome size ^28^. Based on the total number of k-mers obtained, the Argane genome size was calculated to be approximately 573 Mb and 615 Mb, for 21- and 25-mers respectively, using the following formula: total number of k-mer / Peak depth. The double peak of k-mer distribution indicates heterozygosity whose rate is estimated to be 1.58 % and the duplicated fraction of the genome is estimated to be 3,19% ( Figure 2). The estimated genome size seems to be credible compared to the ones of four other Sapotaceae family members. In fact, according to the Plant DNA c-values Database, the genome sizes of these four species ranged from 273 Mb in *Mimusops elengi* L. (c = 0.28 pg) to 2,513 Mb in *Isonandra villosa* L. (c = 2.57 pg). The other two species are *Planchonella eerwah* (c = 0.54 pg, 528 Mb) and *Madhuca longifolia* (c = 0.99 pg, 968 Mb).

### Genome assembly and evaluation

Prior to assembly, Illumina raw reads and PacBio CCS reads were trimmed for adaptor removal using *bbduk.sh* from BBmap suite ( https://github.com/BioInfoTools/BBMap). Short and long reads were assembled following a hybrid approach using MaSuRCA assembler v.3.2.2 ^29^. The initial assembly consists of 671,690,540 bp composed of 82,183 contigs with the largest size being 422,848 bp and an N50 of 43,654 bp. The very few contigs (8) with length less than 200 bp were filtered out and the remaining contigs were scaffolded into 75,327 scaffolds totaling 670,096,797 bp; the N50 reached 49,916 bp and the assembly accounted for 2,982,868 Ns with 445.14 Ns per 100 kb ( Table 2). The scaffolding was done using initial contigs and implemented in MaSuRCA v3.2.4 assembler script using Celera Assembler v8.3. The GC content was estimated to be 33%. The assembly was screened by VecScreen to look for and remove remaining vector contamination. Based on the VecSreen report, contigs containing mitochondrial/chloroplast were also removed. Trimmed PE reads were mapped on the final assembly using CLC genomics (v11.0, CLCbio, Arhus, Denmark) with 0.8 in length and 0.9 in sequence similarity. In total, 94% of the reads were mapped against the Argane genome. The 6% reads that were unmapped may result from the stringency of mapping criteria used.

**Table 2.** Statistics of the *Argania* spinosa genome assembly.

	Number	Total size (bp)	N50 (bp)	Largest (bp)
Contigs	82,183	671,690,540	43,654	422,848
Scaffolds ≥ 200 bp	75,327	670,096,797	49,916	422,848

N50 size defined as the value N such that at least 50% of the genome is covered by scaffolds of size N or larger.

The difference between the genome size estimation and assembly size may be due to the use of parameters excluding extremely high frequency *k-*mers. They often represent organelle sequences, eventual contaminants inflating the genome size estimation ^30^, or the high-frequency of repetitive regions found in plant genomes. Furthermore, the genome is highly heterozygous and different allelic regions would inflate assembly size. To assess the completeness of the final assembly, a Benchmarking Universal Single-Copy Orthologs (BUSCO) v3 software approach was used with “embryophyta_odb9” lineage-specific orthologous groups ^31^. Thus from a total of 1,440 BUSCO genes, 1291 genes (89%) were complete (1179 in single copy and 112 duplicated), 62 genes (4.3%) were represented partially while 87 genes (6%) were missing” from the assembly.

## Conclusions

This draft genome assembly is a first step towards a global and integrative omics strategy for exhaustive characterization of the Argane tree. In particular, future work will focus on structurally annotating the genome using predictive tools and transcriptome analysis. Other future work will focus on functional gene annotation, finding evidence for genome duplication and comparative genome evolution. A reliable annotation is highly dependent on transcriptomic data, analysis and research. Sequencing of different parts and developmental stages of the Argane transcriptome is ongoing. The metabolome, and analysis of Argane oil biosynthesis, as well as the tree’s microbiome should also be analyzed. To this end, and in order to coordinate the strong interests of the Plant Genomics community for this precious tree, the International Argane Genome Consortium (IAGC) and a resource website has been created ( www.arganome.org).

## Data availability

All of the *A. spinosa* datasets can be retrieved under BioProject accession number PRJNA294096: http://identifiers.org/bioproject:PRJNA294096. The raw reads are available at NCBI Sequence Reads Archive under accession number SRP077839: http://identifiers.org/insdc.sra:SRP077839. The complete genome sequence assembly project has been deposited at GenBank under accession number QLOD00000000: http://identifiers.org/ncbigi/GI:1408199612. Data can also be retrieved via the International Argane Genome Consortium (IAGC) website: http://www.arganome.org.

## Author information

Slimane Khayi and Nour Elhouda Azza are co-first authors; Rachid Mentag and Hassan Ghazal contributed equally as supervisors.
