# Supplementary material for: First draft genome assembly of the Argane tree ( Argania spinosa)
Source: F1000Res. 2020 May 4;7:1310. Originally published 2018 Aug 17. [Version 2] doi: 10.12688/f1000research.15719.2 (PMC7238458; doi:10.12688/f1000research.15719.2)
Supplement: Peer review history

# Peer review: Reviewer response for version 2

**DOI:** 10.5256/f1000research.25962.r63017

**Published:** 2020-05-19

**Authors:** Amit Sinha

**Affiliations:** 1 New England Biolabs Inc., Ipswich, MA, USA

The authors have revised the manuscript and have addressed all the concerns.

 It is hoped that the future updates to the draft genome will include the annotation of repeat regions (TE, retrotransposons, etc) and gene predictions and annotations.

Is the work clearly and accurately presented and does it cite the current literature?

Yes

If applicable, is the statistical analysis and its interpretation appropriate?

Yes

Are all the source data underlying the results available to ensure full reproducibility?

Yes

Is the study design appropriate and is the work technically sound?

Yes

Are the conclusions drawn adequately supported by the results?

Yes

Are sufficient details of methods and analysis provided to allow replication by others?

Yes

Reviewer Expertise:

Genome sequencing and assembly, Comparative genomics.

I confirm that I have read this submission and believe that I have an appropriate level of expertise to confirm that it is of an acceptable scientific standard.

# Peer review: Reviewer response for version 2

**DOI:** 10.5256/f1000research.25962.r63016

**Published:** 2020-05-04

**Authors:** Granger Sutton

**Affiliations:** 1 J. Craig Venter Institute, 9704 Medical Center Drive, Rockville, MD 20850, USA

The authors addressed my concerns with their revisions.

Is the work clearly and accurately presented and does it cite the current literature?

Yes

If applicable, is the statistical analysis and its interpretation appropriate?

Not applicable

Are all the source data underlying the results available to ensure full reproducibility?

Yes

Is the study design appropriate and is the work technically sound?

Yes

Are the conclusions drawn adequately supported by the results?

Yes

Are sufficient details of methods and analysis provided to allow replication by others?

Yes

Reviewer Expertise:

Whole genome shotgun assembly, pan-genome analysis

I confirm that I have read this submission and believe that I have an appropriate level of expertise to confirm that it is of an acceptable scientific standard.

# Peer review: Reviewer response for version 1

**DOI:** 10.5256/f1000research.17156.r38276

**Published:** 2018-09-18

**Authors:** Granger Sutton

**Affiliations:** 1 J. Craig Venter Institute, 9704 Medical Center Drive, Rockville, MD 20850, USA

In the article “First draft genome assembly of the Argane tree ( *Argania spinosa*)”, the authors present the draft assembly of the Argane tree using a combination of Illumina and PacBio reads. An argument for the importance of the Argane tree  to the agriculture and economy of the endemic region is given. The assertion that “the genome may enable genome-assisted cultivar breeding, and provide a better understanding of important metabolic pathways and their underlying genes for both cosmetic and pharmacological purposes” seems reasonable based on the use of previous genome sequences for important agricultural resources. This article does not claim much beyond providing a reasonable draft genome for *A. spinosa*. The primary evidence for reasonable quality is three-fold: a k-mer analysis of genome size using Jellyfish, 94% of Illumina reads mapping back to the assembly, and a BUSCO analysis showing 89% full length and 4.3% partial length gene matches for these single copy genes. This is probably sufficient to claim reasonable quality and the usefulness of the genome for downstream research given the contiguity shown by the N50 contig size.

 The authors do miss an opportunity to look more closely at the haplotype separation that may be occurring as they speculate possibly leading to a somewhat larger genome size than indicated by Jellyfish. I’m not sure why the number of BUSCO genes with more than one full or partial length match was not given – perhaps there were none? The implication that heterozygosity is uniform “The double peak of *k*-mer distribution indicates heterozygosity whose rate is estimated to be 1.58%” seems unfounded without more evidence nor is it clear that is the model resulting in this estimate.

 Lastly the grammar would benefit from editing. For example, “A reliable annotation is highly dependent on transcriptomic research, and sequencing of Argane transcriptome analysis of different parts and developmental stages or the plant is ongoing.” Might be better as “A reliable annotation is highly dependent on transcriptomic data, analysis and research. Sequencing of different parts and developmental stages of the Argane transcriptome is ongoing.”.

Is the work clearly and accurately presented and does it cite the current literature?

Yes

If applicable, is the statistical analysis and its interpretation appropriate?

Not applicable

Are all the source data underlying the results available to ensure full reproducibility?

Yes

Is the study design appropriate and is the work technically sound?

Yes

Are the conclusions drawn adequately supported by the results?

Yes

Are sufficient details of methods and analysis provided to allow replication by others?

Yes

Reviewer Expertise:

Whole genome shotgun assembly, pan-genome analysis

I confirm that I have read this submission and believe that I have an appropriate level of expertise to confirm that it is of an acceptable scientific standard.

# Response

**Published:** 2020-04-21

**Authors:** Rachid Mentag

**Affiliations:** INRA Morocco, Morocco

**Answers to Reviewer #2**

 1- The authors do miss an opportunity to look more closely at the haplotype separation that may be occurring as they speculate possibly leading to a somewhat larger genome size than indicated by Jellyfish. I’m not sure why the number of BUSCO genes with more than one full or partial length match was not given – perhaps there were none? The implication that heterozygosity is uniform “The double peak of k-mer distribution indicates heterozygosity whose rate is estimated to be 1.58%” seems unfounded without more evidence nor is it clear that is the model resulting in this estimate. 

**Answer**. We agree with the reviewer. The manuscript has been corrected:

- “..which showed that the assembly contained 89% (1271 genes) of complete and 4.3% (62 genes) of partial sequences that were Arabidopsis orthologs.” has been replaced by “. Thus, from a total of 1,440 BUSCO genes, 1291 genes (89%) were complete (1179 in single copy and 112 duplicated), 62 genes (4.3%) were represented partially while 87 genes (6%) were missing” from the assembly.

2- Lastly the grammar would benefit from editing. For example, “A reliable annotation is highly dependent on transcriptomic research, and sequencing of Argane transcriptome analysis of different parts and developmental stages or the plant is ongoing.” Might be better as “A reliable annotation is highly dependent on transcriptomic data, analysis and research. Sequencing of different parts and developmental stages of the Argane transcriptome is ongoing.”. 

**Answer**. We agree with the reviewer. The manuscript has been corrected:

-  " A reliable annotation is highly dependent on transcriptomic research, and sequencing of Argane transcriptome analysis of different parts and developmental stages or the plant is ongoing" has been replaced by " A reliable annotation is highly dependent on transcriptomic data, analysis and research. Sequencing of different parts and developmental stages of the Argane transcriptome is ongoing.”

# Peer review: Reviewer response for version 1

**DOI:** 10.5256/f1000research.17156.r38275

**Published:** 2018-09-17

**Authors:** Amit Sinha

**Affiliations:** 1 New England Biolabs Inc., Ipswich, MA, USA

In the current manuscript, the authors present the first draft genome assembly for the argan tree *Argania spinosa*, a tree with significant ecological as well as commercial importance.

 The authors have used a hybrid assembly approach, combing data from both Illumina short-reads and PacBio long-reads technology. Use of such hybrid approaches is welcome, as they can combine the  advantages offered by different technological platforms.

 Based on a k-mer analysis, the authors also provide a reasonable estimate of the genome-size and the level of heterozygosity. These are important metrics to report, as they can guide the methods to be used for genome assembly, as well as for interpretation of the assembled genome sequence. The draft genome has been satisfactorily assembled, using state of the art computational methods, and it contains 89% of the predicted core genes as defined in the BUSCO software.

 It is hoped that in their future studies, the authors will follow up with more improvements to the draft genome, including gene annotations using transcriptomic data.

 Overall, manuscript provides valuable information and sequence resources that will be useful to the scientific community.  I have only 1 major and 6 minor comments/suggestions that I think the authors should be able to address.

**Major reservation:**

 1. Both Illumina as well as PacBio reads have been processed and trimmed before being used as an input to the MaSuRCA software. However, the instructions for the MaSuRCA software explicitly ask for **NOT** using any third-party tools, or performing any trimming, cleaning or error correction on Illumina reads, as it will most likely lead to a deteriorated assembly  (See https://github.com/alekseyzimin/masurca#overview). It is therefore strongly suggested that the authors try an assembly with the raw Illumina reads directly, as it might lead to a better assembly. If such an analysis was already performed but the assembly was worse than the current version, please provide this information in the manuscript.

**Minor comment/suggestion:**

 1. For PacBio data, please specify the form of processed reads that were input to the MaSuRCA software: were they the raw PacBio reads, or the Circular Consensus Sequence  (CCS) reads?

 2. The authors report that the genomic DNA was size-selected for fragments above 2kb. What was the approximate maximum size of input DNA for PacBio library preparation. Also, what was the maximum size of PacBio reads (e.g after the CCS step) ?

 3. The raw coverage obtained from Illumina data is reported as 160X. But as per my calculations, based on number of trimmed bases in table 1 and a estimated genome size of 573 Mb, I obtain 135,539,587,270 / 573,000,000 =  236X. Please correct or explain further.

 4. In addition to the observed heterozygosity, the repeat content of the genome could be an important reason for a fragmented assembly. The k-mer analysis can also be used to estimate the % of genome that is comprised of repeats. It will be great if this estimate is provided.

 5. For the BUSCO analysis, the authors report using the Arabidopsis lineage-specific orthologous groups. However, from the BUSCO manual, the only valid value for the lineage parameter seems to be “embryophyta_odb9”, which internally uses Arabidopsis hmms for Augustus-based gene predictions, but the member proteins are form multiple species. If this is the lineage parameter the authors used, please specifiy it as “embryophyta_odb9” rather than Arabidopsis, or provide more details on how the Arabidopsis BUSCO groups were determined.

 6. The genome sequences of the organelles mitochondria and chloroplast are also very important in investigating the biology of any species, and also serve as important resource for evolutionary and phylogenetic studies. Therefore, it will be great if instead of completely removing the organellar genomes, the authors provide them as separate set of sequences, in separate files and ideally as separate GenBank entries.

Is the work clearly and accurately presented and does it cite the current literature?

Yes

If applicable, is the statistical analysis and its interpretation appropriate?

Yes

Are all the source data underlying the results available to ensure full reproducibility?

Yes

Is the study design appropriate and is the work technically sound?

Yes

Are the conclusions drawn adequately supported by the results?

Yes

Are sufficient details of methods and analysis provided to allow replication by others?

Yes

Reviewer Expertise:

NA

I confirm that I have read this submission and believe that I have an appropriate level of expertise to confirm that it is of an acceptable scientific standard, however I have significant reservations, as outlined above.

# Response

**Published:** 2019-07-15

**Authors:** Hassan Ghazal

**Affiliations:** UMP, Morocco

1. Both Illumina as well as PacBio reads have been processed and trimmed before being used as an input to the MaSuRCA software. However, the instructions for the MaSuRCA software explicitly ask for **NOT** using any third-party tools, or performing any trimming, cleaning or error correction on Illumina reads, as it will most likely lead to a deteriorated assembly  (See https://github.com/alekseyzimin/masurca#overview). It is therefore strongly suggested that the authors try an assembly with the raw Illumina reads directly, as it might lead to a better assembly. If such an analysis was already performed but the assembly was worse than the current version, please provide this information in the manuscript.

**Response**:

 Both illumina and PacBio reads have been trimmed only for adaptor removal.  No quality trimming was performed. (The manuscript has been corrected):

1. “Quality-filtered reads from the Illumina” has been replaced by “ **Trimmed reads from the Illumina**”
2. “These data was trimmed of adapters and low-quality sequences,…” has been replaced by **“These data was trimmed of adapters,…**”
3. “Prior to assembly, Illumina and PacBio raw reads were trimmed for quality and adaptor removal using *bbduk.sh”* has been replaced by ***“*** **Prior to assembly, Illumina raw reads and PacBio CCS reads were trimmed for adaptor removal using *bbduk.sh”***

 

**Minor comments/suggestions:** 

 1. For PacBio data, please specify the form of processed reads that were input to the MaSuRCA software: were they the raw PacBio reads, or the Circular Consensus Sequence  (CCS) reads? **Response:**

 CCS PacBio reads were processed within MaSuRCA software

 2. The authors report that the genomic DNA was size-selected for fragments above 2kb. What was the approximate maximum size of input DNA for PacBio library preparation. Also, what was the maximum size of PacBio reads (e.g after the CCS step) ? 

**Response:**

 DNA extracted from the lyophilized leaves was divided into HMW and LMW fractions, and each was used separately to make PacBio libraries. The best PacBio SMRT cell yielded 4G of data on 163,000 spots, with an average read size of 24,468 bases. 

 3. The raw coverage obtained from Illumina data is reported as 160X. But as per my calculations, based on number of trimmed bases in table 1 and an estimated genome size of 573 Mb, I obtain 135,539,587,270 / 573,000,000 = 236X. Please correct or explain further.

**Response:**

 We agree with the reviewer. The manuscript have been corrected:“These data was trimmed of adapters and low-quality sequences, yielding a clean set of 936,053,040 reads, representing 160× genome coverage,” has now been replaced by **“These data was trimmed of adapters and low-quality sequences, yielding a clean set of 936,053,040 reads, representing 236x genome coverage**,”

 4. In addition to the observed heterozygosity, the repeat content of the genome could be an important reason for a fragmented assembly. The k-mer analysis can also be used to estimate the % of genome that is comprised of repeats. It will be great if this estimate is provided.

**Response:**

 Based on the 21-mer distribution analysis, the duplicated fraction of the genome is estimated to be 3,19%. The revised version of the manuscript has been corrected:

 “The double peak of k-mer distribution indicates heterozygosity whose rate is estimated to be 1.58 % ( Figure 2).” Has been replaced by **“The double peak of k-mer distribution indicates heterozygosity whose rate is estimated to be 1.58 % and the duplicated fraction of the genome is estimated to be 3,19% ( Figure 2).”**

 5. For the BUSCO analysis, the authors report using the Arabidopsis lineage-specific orthologous groups. However, from the BUSCO manual, the only valid value for the lineage parameter seems to be “embryophyta_odb9”, which internally uses Arabidopsis hmms for Augustus-based gene predictions, but the member proteins are form multiple species. If this is the lineage parameter the authors used, please specifiy it as “embryophyta_odb9” rather than Arabidopsis, or provide more details on how the Arabidopsis BUSCO groups were determined.

**Response:**

 We agree. The lineage parameter used is “embryophyta_odb9”. (The manuscript has been corrected):

 “To assess the completeness of the final assembly, a Benchmarking Universal Single-Copy Orthologs (BUSCO) v3 software approach was used with Arabidopsis lineage-specific orthologous groups 31,” has been replaced by **“To assess the completeness of the final assembly, a Benchmarking Universal Single-Copy Orthologs (BUSCO) v3 software approach was used with “embryophyta_odb9” lineage-specific orthologous groups 31,**”

 6. The genome sequences of the organelles mitochondria and chloroplast are also very important in investigating the biology of any species, and also serve as important resource for evolutionary and phylogenetic studies. Therefore, it will be great if instead of completely removing the organellar genomes, the authors provide them as separate set of sequences, in separate files and ideally as separate GenBank entries. 

**Response:**

 We agree with the reviewer. The work is in progress and we plant to publish the organellar genomes soon in a separate manuscript.

# Response

**Published:** 2020-04-21

**Authors:** Rachid Mentag

**Affiliations:** INRA Morocco, Morocco

**Answers to Reviewer #1**

**Major reservation:**

 1. Both Illumina as well as PacBio reads have been processed and trimmed before being used as an input to the MaSuRCA software. However, the instructions for the MaSuRCA software explicitly ask for **NOT** using any third-party tools, or performing any trimming, cleaning or error correction on Illumina reads, as it will most likely lead to a deteriorated assembly  (See https://github.com/alekseyzimin/masurca#overview). It is therefore strongly suggested that the authors try an assembly with the raw Illumina reads directly, as it might lead to a better assembly. If such an analysis was already performed but the assembly was worse than the current version, please provide this information in the manuscript.

**Answer.**We agree with the reviewer. Both illumina and PacBio reads have been trimmed only for adaptor removal. No quality trimming was performed. The manuscript has been corrected:

  

- “Quality-filtered reads from the Illumina” has been replaced by “Trimmed reads from the Illumina”
- “These data was trimmed of adapters and low-quality sequences,…” has been replaced by “These data was trimmed of adapters,…”
- “Prior to assembly, Illumina and PacBio raw reads were trimmed for quality and adaptor removal using bbduk.sh” has beenreplaced by “Prior to assembly, Illumina raw reads and PacBio CCS reads were trimmed for adaptor removal using bbduk.sh” 

**Minor comment/suggestion:**

 1- For PacBio data, please specify the form of processed reads that were input to the MaSuRCA software: were they the raw PacBio reads, or the Circular Consensus Sequence  (CCS) reads? 

**Answer**. CCS PacBio reads were processed within MaSuRCA software. The manuscript has been corrected:

  

- “Prior to assembly, Short and long reads were…” has been replaced by “Prior to assembly, Illumina raw reads and PacBio CCS reads were …”

2- The authors report that the genomic DNA was size-selected for fragments above 2kb. What was the approximate maximum size of input DNA for PacBio library preparation. Also, what was the maximum size of PacBio reads (e.g after the CCS step) ? 

**Answer**.DNA extracted from the lyophilized leaves was divided into HMW and LMW fractions, and each was used separately to make PacBio libraries. The best PacBio SMRT cell yielded 4G of data on 163,000 spots, with an average read size of 24,468 bases.

 3- The raw coverage obtained from Illumina data is reported as 160X. But as per my calculations, based on number of trimmed bases in table 1 and a estimated genome size of 573 Mb, I obtain 135,539,587,270 / 573,000,000 = 236X. Please correct or explain further. 

**Answer**.We agree with the reviewer. The manuscript have been corrected:

  

- “….yielding a clean set of 936,053,040 reads, representing 160× genome coverage,” has now been replaced by “…yielding a clean set of 936,053,040 reads, representing 236x genome coverage,”

 

 4- In addition to the observed heterozygosity, the repeat content of the genome could be an important reason for a fragmented assembly. The k-mer analysis can also be used to estimate the % of genome that is comprised of repeats. It will be great if this estimate is provided. 

**Answer**.Based on the 21-mer distribution analysis, the duplicated fraction of the genome is estimated to be 3,19%. The revised version of the manuscript has been corrected:

  

- “The double peak of k-mer distribution indicates heterozygosity whose rate is estimated to be 1.58 % ( Figure 2).” Has been replaced by “The double peak of k-mer distribution indicates heterozygosity whose rate is estimated to be 1.58 % and the duplicated fraction of the genome is estimated to be 3,19% ( Figure 2).”

 

 5- For the BUSCO analysis, the authors report using the Arabidopsis lineage-specific orthologous groups. However, from the BUSCO manual, the only valid value for the lineage parameter seems to be “embryophyta_odb9”, which internally uses Arabidopsis hmms for Augustus-based gene predictions, but the member proteins are form multiple species. If this is the lineage parameter the authors used, please specifiy it as “embryophyta_odb9” rather than Arabidopsis, or provide more details on how the Arabidopsis BUSCO groups were determined. 

**Answer**. We agree with the reviewer. The lineage parameter used was “embryophyta_odb9”. The manuscript has been corrected:

  

- “To assess the completeness of the final assembly, a Benchmarking Universal Single-Copy Orthologs (BUSCO) v3 software approach was used with Arabidopsis lineage-specific orthologous groups...” has been replaced by “To assess the completeness of the final assembly, a Benchmarking Universal Single-Copy Orthologs (BUSCO) v3 software approach was used with “embryophyta_odb9” lineage-specific orthologous groups...”

 

 6- The genome sequences of the organelles mitochondria and chloroplast are also very important in investigating the biology of any species, and also serve as important resource for evolutionary and phylogenetic studies. Therefore, it will be great if instead of completely removing the organellar genomes, the authors provide them as separate set of sequences, in separate files and ideally as separate GenBank entries. 

**Answer**.We agree with the reviewer. The work is in progress and we plan to publish the organellar genomes soon in separate manuscripts.
